# A prospective case series for a minimally invasive internal fixation device for anterior pelvic ring fractures

**DOI:** 10.1186/s13018-016-0468-9

**Published:** 2016-11-08

**Authors:** Wayne Hoskins, Andrew Bucknill, James Wong, Edward Britton, Rodney Judson, Kellie Gumm, Roselyn Santos, Rohan Sheehy, Xavier Griffin

**Affiliations:** 1 Department of Orthopaedic Surgery, Royal Melbourne Hospital, Grattan St, Parkville, VIC 3052 Australia; 2 Faculty of Medicine, Dentistry and Health Sciences, The University of Melbourne, Parkville, Victoria Australia; 3 Trauma Service, Royal Melbourne Hospital, Parkville, Victoria Australia; 4 Nuffield Department of Orthopaedics, University of Oxford, Oxford, UK; 5 John Radcliffe Hospital, Oxford University Hospitals NHS Trust, Headington, Oxford, UK

**Keywords:** Pelvis, Fractures, Bones, Fracture fixation, Wounds and injuries

## Abstract

**Background:**

External fixation is commonly used as a means of definitive fixation of pelvic fractures. Pin site infection is common, with some cases of osteomyelitis and inpatient nursing can be challenging. The aim of this study is to report the outcomes and complications of an alternative minimally invasive technique, known as INFIX, utilising spinal pedicle screws inserted into the supra-acetabular bone and connected by a subcutaneous rod.

**Methods:**

A single-centre prospective case series was performed. The primary outcome measures were fracture stability and displacement at time of implant removal and intra- and post-operative complications.

**Results:**

Twenty-one patients were recruited, with 85.7 % of fractures being lateral compression type. Mean follow-up was 342 days. Mean application time was 51 min (range 44–65). Nineteen were removed electively, with mean time to removal 109 days. All cases were stable with no displacement. Two cases were removed emergently, one due to wound infection and the other due to lateral femoral cutaneous nerve neuropathic pain. Twelve patients sustained a lateral femoral cutaneous nerve palsy, with 20/42 nerves being affected. Improvement in all lateral femoral cutaneous nerve symptoms were reported with removal. Nine patients developed asymptomatic heterotopic ossification, and there were three deep infections and one symptomatic due to the bar.

**Conclusions:**

Minimally invasive internal fixation with the INFIX for anterior pelvic ring fractures is an alternative to anterior external fixation. However, a higher rate of lateral femoral cutaneous nerve palsy is noted, and the implant is not well tolerated by all patients. Further studies are required to define fracture types and patients best suited to the technique and how LFCN complications may be minimised.

**Trial registration:**

ACTRN12616001421426. Registered 12 October 2016. Retrospectively registered.

## Background

Anterior pelvic ring fixation may be achieved using multiple techniques. Minimally invasive techniques are increasingly reported in the literature as an alternative to traditional external fixators [1]. External fixation has typically been used temporarily for unstable pelvic fractures in damage control situations with haemodynamic instability, although their application can be definitive management [2, 3]. External fixation has a number of disadvantages, with pin site infection being reported in 2–50 % and osteomyelitis in 0–7 % [2, 4, 5]. Other disadvantages reported in the literature include aseptic loosening in 0–19 %, loss of reduction in 0–33 % and challenges with nursing care, particularly in obese patients [2, 6].

The INFIX or “Pelvic Bridge” is a new technique that that was initially developed for use in obese patients and uses the principles of external fixation [7]. It involves insertion of supra-acetabular pedicle screws connected by a subcutaneous cobalt-chromium contoured rod. The large diameter pedicle screws anchored in the strong bone allow pelvic reduction manoeuvres and compression at the fracture site. Such principles make INFIX an attractive surgical treatment modality, particularly for comminuted fractures of the anterior pelvic ring. A second procedure is required for the removal of the implant at a minimum of 3-month time frame, which is most commonly described in the literature [5, 8]. Biomechanical studies have shown that the minimally invasive INFIX has superior stability to external fixation, due to the shorter lever arm of the construct [9]. However, compared with internal fixation, it has less stability and stiffness [9].

In a recent randomised controlled trial comparing minimally invasive pelvic surgery with external pelvic fixation, of the 23 patients who met the inclusion criteria, 12 (48 %) of patients recruited refused participation because of the possibility of external fixation [1].

There are few clinical reports of outcomes following INFIX. Level 4 evidence available as case series have reported positive initial results with loss of reduction occurring in 0–2 %, revision surgery in 0–7 % and wound infection in 0–4 % [5, 8, 10]. The most common complication reported is lateral femoral cutaneous nerve palsy, reported to occur temporarily in 0–30 % and permanently in 0–1 % [5, 8]. Heterotopic ossification has been reported in 0–25 % [5, 8].

Indications for the INFIX are not fully defined, and there is no agreement in the literature as to what fractures are best suited for their application. Some authors report as an indication for use when a traditional pelvic external fixator may have been used definitively [8]. Others use it for rotational or vertically unstable pelvic fractures: APC2, APC3, LC2, LC3 [5] or for AO Orthopaedic Trauma Association type C injuries [8]. However, the classification of pelvic fractures is unreliable, even amongst pelvic surgeons, with low inter-examiner reliability [11], making the challenge of defining and reporting suitable fracture types vexed. The failure of the INFIX in the management of a morbidly obese patient with pubic diastasis [12] would suggest a limitation of the technique with open-book pelvic fractures which would be better managed with a stiffer and more stable internal fixation construct [9]. We report a single-centre prospective case series of the outcomes and complications associated with the INFIX technique.

## Methods

A single-centre prospective case series at a single Level 1 Trauma Centre was performed from February 2013 until July 2014. The indication for the use of INFIX was a fracture that the primary surgeon deemed as an unstable anterior ring fracture, typically with fracture site comminution. Fractures were classified using the Young-Burgess classification [13]. Patients were excluded in damage control situations with haemodynamic instability, open wounds, pubic diastasis injury, in paediatric and/or very low body mass index and the presence of hernias at the site surgical site. The INFIX was not used for fixation of pubic symphysis injury. The case series was commenced after a 10-patient learning curve by the primary surgeon. Participants were excluded if there was not a minimum 6-month follow-up. The primary outcome measures were stability and fracture displacement at time of implant removal (assessed by manual stress of the pelvic ring under anaesthesia) and intra- and post-operative complications. Data were collected from the Royal Melbourne Hospital Trauma Registry. Descriptive statistics were used to analyse results.

### Surgical technique

If required, stability of the posterior pelvic ring was achieved prior to application of the INFIX. For application of the INFIX, a 3-cm vertical or oblique incision was made lateral to the interval between sartorius and tensor fascia lata (TFL), as per the Smith-Petersen or anterior approach to the hip joint [14]. The fascia of TFL was incised and blunt dissection continued to the anterior inferior iliac spine (AIIS) between the belly of TFL and sartorius, reflecting the fascia medially with sartorius to protect the lateral femoral cutaneous nerve (LFCN). A 4.3-mm drill was used to open the bony safe corridor for the screws, with an entry point at the AIIS, using fluoroscopy to confirm the accurate placement on Judet views of the iliac wing. The drills were exchange for guide wires and the corridors tapped to 9 mm over the guide wire. Cannulated pedicle screws (DePuy SAI Viper, Warsaw, Indiana) (10 mm × 100 mm) were inserted, also over the guide wires. Screw position was confirmed with fluoroscopy. The screw head was made to sit such that the connecting bar was subcutaneous and superficial to the fascia of the abdominal wall. A 5.5-mm cobalt-chromium bar was contoured then placed subcutaneously. The rod was connected to the screw heads and locked at one end. Reduction of the anterior pelvic ring injury was achieved by compression or distraction of the rod prior to locking of the remaining screw head.

## Results

Twenty-one patients were recruited with a mean age of 39 (range 16–73). Nineteen operations were performed for acute pelvic ring fractures and two for salvage procedures followed failed open reduction and internal fixation of anterior ring fractures. Mean follow-up time was 342 days (range 182–537). Participants had a mean injury severity score (ISS) of 23 (range 5–43). Sixteen patients had an ISS greater than 12, defined as major trauma by the Victorian State Trauma Service [15]. Sixteen participants were admitted directly to Royal Melbourne Hospital, five arrived via inter-hospital transfer. Most injuries were a result of motor vehicle accidents: car accidents (*n* = 7), pedestrian versus car (*n* = 6), falls greater than 1 m (*n* = 3), motorcycle accidents (*n* = 3) and bicycle versus car accident (*n* = 2). Most fractures were LC1 (57.1 %, *n* = 12), followed by LC2 (28.6 %, *n* = 6), APC3 (9.5 %, *n* = 2) and APC2 (4.8 %, *n* = 1).

The mean time to surgery was 5 days (range 0–19, median 5). Mean application time from skin incision to skin closure with sutures was 51 min (range 44–65). Nineteen of the 21 patients had additional posterior ring fixation. Mean length of stay was 18.9 days (range 5–97, median 14). Nine of the 21 patients were admitted to the Intensive Care Unit for a mean of 6.7 days (range 3–25).

Nineteen of the 21 INFIX’s were removed electively, with a mean time to removal of 109 days. All cases were stable on examination under anaesthesia with no displacement. Two cases were removed emergently, one due to wound infection at day 56 and the other due to LFCN neuropathic pain at day 62. Both of these cases were stable on examination under anaesthesia.

Twelve of the 21 patients sustained a LFCN palsy, with 20/42 nerves being affected. Two patients developed neuropathic pain requiring management with neuropathic analgesia. One of these patients had earlier than planned removal of the INFIX. Both patients’ symptoms resolved following removal of the implant. Improvement in all LFCN symptoms was reported with removal although all symptoms persisted to some extent with follow-up.

Nine of the 21 patients developed heterotopic ossification noted at time of implant removal, occurring to 14/42 sides with only one being symptomatic. There were 3/42 deep infections, 2/21 non-fatal pulmonary embolisms and 1/21 patient was symptomatic due to the bar, with complete resolution after removal.

## Discussion

We have reported 21 patients over 18 months with an approximate 1-year follow-up in a single major trauma centre. We found that the INFIX procedure yielded excellent results for fracture reduction and stability. However, the bar was poorly tolerated in nearly 10% of patients, there was a high proportion of LFCN palsy and heterotopic ossification, and a second procedure was required for removal of the implant. There was a short learning curve for the technique, which commenced prior to the commencement of the study and short operative time.

Given the similarities of our surgical technique to that previously reported, it is unclear why our case series reported a much higher rate of complications, in particular LFCN palsy and heterotopic ossification. The largest series found approximately one third of patients to suffer LFCN irritation and one third to suffer heterotopic ossification [5]. However, we found the heterotopic ossification around the implants at removal and like the Vaidya et al. [5] series, it did not interfere with the function or cause any symptoms or complicate implant removal. Despite this, due consideration should be given to the use of prophylactic agents to prevent this complication [16].

The LFCN may injure during the approach or from the implant itself. Our surgical approach is very similar to that previously reported in the literature using a 3-cm incision and blunt dissection in the interval between sartorius and TFL to gain access to the AIIS [7, 17]. Our 10-mm diameter pedicle screws were larger than the 7.0-mm screw used by Owen et al. [12] and the 7- to 8.5-mm screws by Vaidya et al. [5]. It may be that the largest screw and head size contributed to a traction neuropraxia, as blunt dissection and care in the approach was excised similarly to Scheyerer et al. [10], who reported no LFCN palsies using a smaller screw. However, a change in screw size may complicate fracture stability. Loss of fixation has been reported in the literature using small screws [5], whereas all cases in our series were stable on examination under anaesthesia with no displacement at time of implant removal. Further efforts should be made to assess the appropriate screw size required to maintain fracture stability and the mechanism of LFCN palsy and how this complication rate can be decreased to improve the benefit: risk ratio of the INFIX technique.

Our surgical technique described is very similar to that previously reported in the literature. Vaidya et al. [7] in their 24 patient case series also reported addressing posterior stability first. Gardner et al. [8] on the other hand reported performing anterior fixation prior to posterior fixation in their 24 patient case series. Vaidya et al. [7] described a screw that sat 15–40-mm proud and a 6 mm subcutaneous contoured rod. Our surgical time of 51 min was comparable to Sheyerer et al. [10], who averaged an operative time of 50 min (range 45–60 min).

Given the low inter-observer reliability for the Tile/AO (kappa 010 to 0.17) and Young-Burgess (kappa 0.09 to 0.21) classification systems amongst experienced pelvic surgeons [11], it remains a challenge to classify fractures when describing operative techniques for pelvic osteosynthesis. Despite this, the vast majority of fractures in this series were lateral compression in nature, followed by anterior-posterior compression. In the largest published series by Vaidya et al. [5], one third of procedures were for lateral compression fracture patterns, one third anterior-posterior compressions fractures and one third vertical shear or combined, which would seem to expand the possible fracture patterns that may be suited for consideration of INFIX. Other series have predominantly used the technique on OTA C type fractures [7, 8]. Scheyerer et al. [10] suggest as indications for the minimally invasive INFIX technique cases of large pelvic defects, multiple or comminuted fractures of the anterior pelvic ring, coagulopathies and history of the previous hip or abdominal surgery. Our series reinforces these indications—predominantly including those patients with comminuted fractures, which render plate fixation technically demanding, or in those patients where open reduction and internal fixation has failed. In contrast to Vaidya et al. [5], we used the technique for LC1 fracture patterns. Controversy exists as to the role of surgical versus non-surgical management of these fracture patterns [18] and the complications associated with INFIX should be considered in the decision-making process. Further study should better ascertain which fracture patterns may be suited to the procedure. In doing so, comparisons of benefits and risks could be made to different methods of fracture fixation and also non-operative management.

## Conclusions

The INFIX is an option for anterior pelvic ring fractures, although there is a high proportion of LFCN palsy. Indications for its use need to be more clearly defined. No single pelvic surgical technique is appropriate for all injury patterns. The INFIX appears a useful adjunct to the previous more commonly performed procedures.

## Abbreviations

AIIS: Anterior inferior iliac spine
AP: Antero-posterior
LC: Lateral compression
LFCN: Lateral femoral cutaneous nerve
TFL: Tensor fascia lata
